# Are the digit ratio (2D:4D) and hand grip strength related to Parkinson disease in elderly males?

**DOI:** 10.1186/s13102-023-00642-2

**Published:** 2023-03-20

**Authors:** Hamid Arazi, Roghayeh Bavafa Birak Olia, Ehsan Eghbali

**Affiliations:** Department of Exercise Physiology, Faculty of Sport Sciences, University of Guilan, P.O. Box: 41635-1438, Rasht, Iran

**Keywords:** 2D:4D, Parkinson disease, Sexual dimorphism, Handgrip strength, Sex hormones

## Abstract

**Background:**

Sex hormones affect the pathogenesis of Parkinson patients and it has been suggested that gender is the most important factor in the development and progression of Parkinson’s disease. Studies have shown that the second to fourth digit ratio (2D:4D) is affected by the prenatal testosterone and estrogen levels and can predict predisposition to disease. In addition, decreased muscle strength in people with Parkinson’s has been repeatedly reported. Hand grip strength (HGS) is a suitable measure to evaluate the musculoskeletal system among the elderly and it is considered as an indicator of the overall strength of the body. This study aimed at investigating the relationship between Parkinson’s disease and HGS and 2D:4D ratio.

**Methods:**

In this study 117 elderly men with Parkinson disease (mean age of 61.66 ± 11.28 years) and 156 healthy control subjects (mean age of 61.86 ± 6.29 years) participated. After determining the level of disability of Parkinson patients by a neurologist (level of disability in the range of 1–4), anthropometric indices (height, weight, length of the second and fourth fingers) and maximum HGS were measured.

**Results:**

Although 2D:4D ratios (right and left hand) of male patients with Parkinson’s disease were higher than those of healthy males, this difference was not statistically significant (P = 0.12, P = 0.40; respectively). Conversely, HGS for the right and left hands of Parkinson patients were significantly lower than those of healthy males (P = 0.02, P = 0.03; respectively). The results showed a significant negative relationship between Parkinson disease and the right and left HGS (R = -0.16, P = 0.005; R = -0.17, P = 0.003; respectively). Parkinson disease had no significant relationship with 2D:4D of the right hand, left hand, mean finger ratio and D_R−L_ 2D:4D (P > 0.05). The regression results showed that the right and left HGS were not able to predict Parkinson disease (P = 0.25, P = 0.16; respectively).

**Conclusion:**

We concluded that HGS was negatively associated with the Parkinson disease, but conversely, 2D:4D may not be a valuable biomarker of elevated risk of Parkinson in elderly males.

## Background

Parkinson is the second chronic neurodegenerative disorder whose main pathophysiology is the destruction of dopamine-containing cells in the basal ganglia [1–3]. Internal nuclei of basal ganglia, deprived of their natural dopaminergic inputs, impair functions leading to abnormal nerve oscillations and synchronization in several basal ganglia-thalamic-cortical circuits [4]. This flow of disorders leads to clinical manifestations of the disease, which including movement disorders such as bradykinesia (slow movements), muscle rigidity, resting tremor, and postural instability. Voluntary movement disorders in Parkinson is specified with a number of specific sensorimotor processing deficits, including general slowness of movement [5], difficulty in performing sequential movements, reliance on sensory input, especially visual input to guide and correct movement and problems in timing and coordination of movements. In addition, hand function control can be completely compromised [6]. Coordination of sensory information with movement planning is very important for proper hand movements. Adjustment of force control is an important parameter for proper hand function which relies on proper activation of the basal ganglia [7]. In Parkinson patients, there is a delay and disruption in the time and speed of isometric force production in both stages of production and release of productive force [8]. Isometric force control in Parkinson patients is also associated with increased variability in grip force by increasing force magnitude or by removal of visual feedback [9].

Reduction of muscle strength in people with Parkinson has been reported repeatedly [10]. Dopaminergic deficits in people with Parkinson reduce stimulation in the motor cortex, which can affect the use of motor unit and lead to muscle weakness [11]. Arm muscle function involves manipulating objects that require the use and integration of muscle activity from the shoulder to the fingers. Evidence suggests that people with Parkinson have weaknesses in some muscle groups, especially the wrists and elbows; even when allowance is made for the slow development of maximal force [12]. A non-invasive and inexpensive method widely used to assess muscle strength is handgrip strength (HGS). This measurement is a reflection of the maximum isometric strength of the muscles of the hand and forearm [13]. HGS is often used as an indicator of measuring muscle strength of the whole body and is considered a practical, fast, easy and independent tool for the observer [14]. However, there is little research on the association of HGS in Parkinson disease; but in general, HGS is lower in people with Parkinson than in healthy people [15].

Gonadal sex hormones, including testosterone and estrogen, are considered crucial in brain development [16]. In addition, sex hormones affect the pathogenesis of Parkinson patients [17] and sex is the most important factor in the development and progression of Parkinson disease. This neurological disease is more common in men than women with an approximate ratio of two to one and the risk of developing it is higher in men [18]. The second to fourth ratio (2D:4D) has been considered reflecting sexual dimorphism (fetal testosterone and estrogen levels) [19]. Study has shown that 2D:4D can predict predisposition to diseases. For example, one studies showed that sex hormone environment in the early development is associated with the risk of developing cancer in life [20]. High prenatal testosterone and low estrogen exposure (i.e., low 2d:4d ratio) are associated with prostate cancer [20–23] and risk of brain tumors [24], or higher 2D:4D ratio increases the risk of coronary heart disease [25]. Moreover, some studies stated that 2D:4D ratio is not associated with testicular cancer [26,27] and gastric cancer stage [28, 29]. It is believed that the digit ratio is fixed in the 14th week of pregnancy and has a negative relationship with fetal testosterone and a positive relationship with fetal estrogen at this time [30]. Researchers have shown that 2D:4D ratio determines the balance between androgen and estrogen [31]. Contradictory results have been obtained between the digit ratios and different diseases. Studies have shown that there is no relationship between the digit ratios and the symptoms of schizophrenia and depression [32, 33]. In contrast, Mackus et al. stated that there was a positive relationship between autism and the digit ratio in men and women, and this relationship was stronger in men [34].

However, the relationship between Parkinson’s disease and the 2D:4D ratio has not been investigated so far, and according to the fact that sex hormones play an important role in Parkinson disease, and the second to fourth finger ratios indirectly indicate a balance between testosterone and estrogen; it seems that digit ratio may be related to the Parkinson disease. In addition, with regard to HGS, it can be noted that this index is suitable as a non-invasive method for assessing general body strength and clinical evaluation of physical function [14] and decreased muscle strength in people with Parkinson has been repeatedly reported. According to the existing mechanisms, it is hypothesized that the digit ratio and HGS is associated with Parkinson disease and it is possible that these indicators can be used for initial assessments of the disease in individuals. Therefore, in this article, researchers seek to investigate the relationship between the second to fourth finger ratios and HGS with Parkinson disease.

## Methods

### Participants

**Fig. 1:**
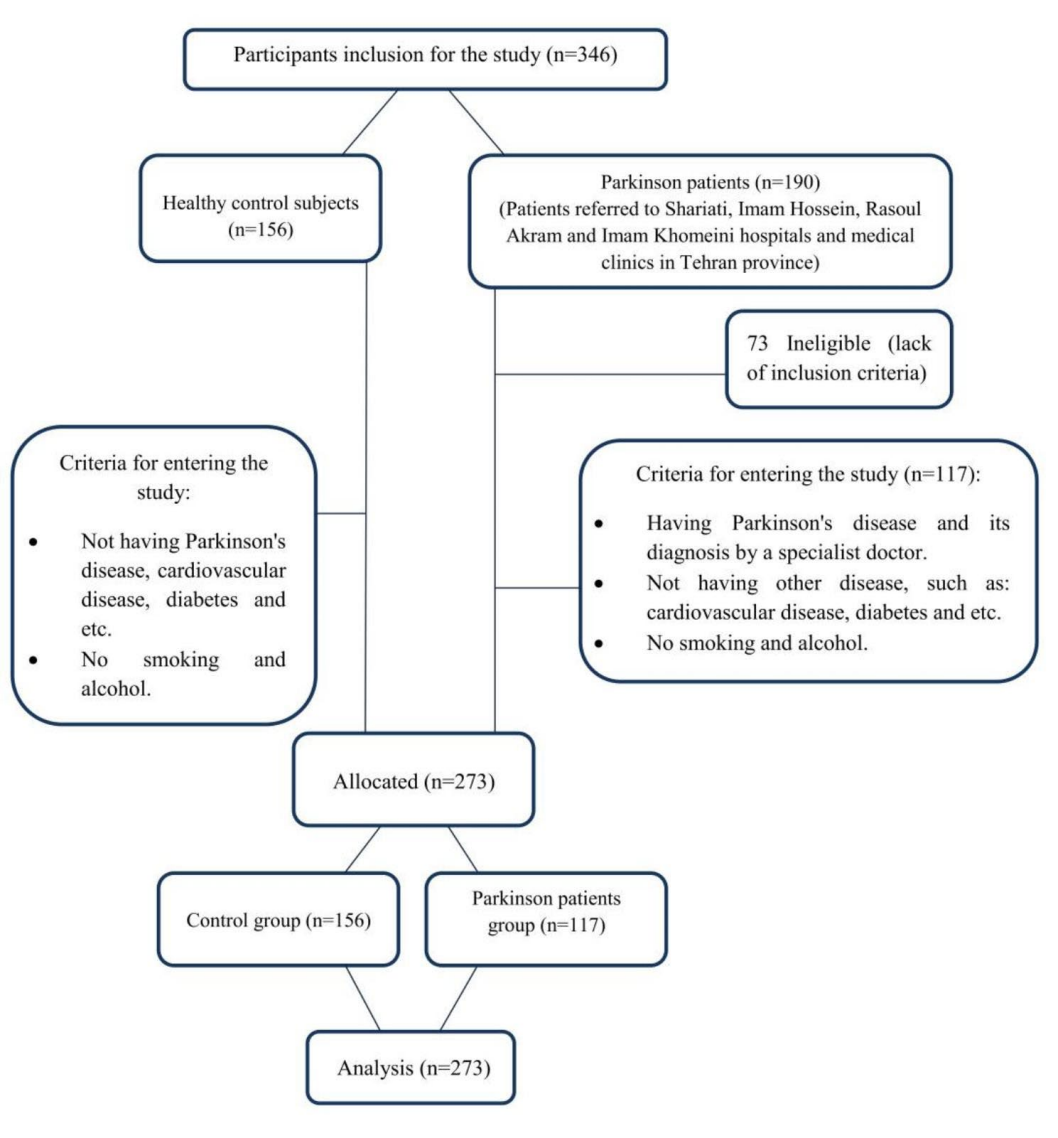
Flow chart of the study

The statistical population of the present study consisted of patients referred to Shariati, Imam Hossein, Rasoul Akram and Imam Khomeini hospitals and medical clinics in Tehran province. A total of 190 male patients with Parkinson volunteered to participate in this study and finally 117 of them (mean age of 61.66 ± 11.28 years) were included in the study. The disease of the participants in this study was confirmed by a neurologist. People with other diseases such as cardiovascular disease, diabetes, etc., or patients who smoked and drank alcohol were excluded from the study (Fig. 1). In addition, 156 healthy men (mean age of 61.86 ± 6.29 years) participated in this study as a control group (not having Parkinson’s disease, cardiovascular disease, diabetes and etc. and they did not smoke or drink alcohol).

After subject’s agreement to participate, the purpose of the research and the procedure were explained to them and the consent form and Beck physical activity questionnaire [35] were completed by the individuals. Then, anthropometric indices including height, weight, second and fourth finger length were measured and how to use a dynamometer to measure maximum HGS was taught and the strength of individuals was evaluated. In addition, the level of disability of patients was determined by a neurologist. This study was approved by the Institutional Review Board at the University of Guilan for the protection of human subjects.

### Digit ratio

The 2D:4D ratio of the right and left hands were calculated according to the method recommended by Manning et al. [30]. The lengths of the index and ring fingers were measured on the palmar surface of the hand from the basal crease proximal to the palm to the tip of the finger using digital calipers (Mitutoyo, model digimatic caliper 500-151-20, Mitutoyo, China) with the accuracy of 0.01 mm (the measurements were repeated for two times) [30]. Re-measurement reliability was high for the first and second 2D:4D (intraclass correlation coefficients (ICC) > 0.95). The digit ratio was obtained by dividing the length of the index finger by the length of the ring finger. Furthermore, the mean hand 2D:4D ratio showed the average value of left hand 2D:4D and right hand 2D:4D, and the right minus left hand 2D:4D (D_R−L_2D:4D) was calculated based on the formula: the D_R−L_ 2D:4D = right hand 2D:4D - left hand 2D:4D [19].

### Handgrip strength

HGS (right and left hand) was measured using a digital dynamometer (Seahan, model SH5003, Seahan Co, South Korea). The participants performed the test in a sitting position on a chair, with their hand flexed to 90° along the vertical axis and their wrists in slight extension (test was repeated 3 times with 30 s rest between trials and the mean value was recorded in kilogram) [36].

### The level of disability of Parkinson patients

The level of disability of Parkinson patients was determined by a neurologist, based on the research of Hoehn and Yahr Scale (level of disability in the range 1–4) [37].

### Statistical analysis

The data were found normal by Kolmogorov–Smirnov test; comparisons of the variables between Parkinson patients and controls groups were performed using the two-way ANOVA test. Partial correlation coefficient test (controlling for age and BMI) was done to discover the correlations between 2D:4D digit ratios and HGS with Parkinson disease. Multiple regression analysis using General Linear Models was also conducted. The effect size was evaluated with Hedges’ g. Cohen (1992) suggested the following interpretation of Hedges’ g effect size: > 0.2 = weak, > 0.5 = medium, > 0.8 = strong effect [38]. The p-value P < 0.001 was regarded as having a significant difference. Data analysis were conducted with SPSS 22.0 software and expressed as mean ± standard deviation (SD).

## Results

The characteristics of the subjects are shown in Table 1. 2D:4D ratios (right and left hand) of male patients with Parkinson were higher than those of healthy males, this difference was not statistically significant (P = 0.12, P = 0.40; respectively). Conversely, HGS right and left hands and physical activity level of Parkinson disease were significantly lower than those of healthy males (P = 0.02, P = 0.03, P < 0.001; respectively). Furthermore, the mean ratio of the fingers and D_R−L_ 2D:4D were not significantly different between the two groups (P = 0.18, P = 0.23, respectively, Table 1).

**Table 1:** Description of participating subjects and differences in 2D:4D ratios and HGS (with p values and effect sizes) between Parkinson patients and controls

Variable	Mean ± SD		P	Effect size
	Parkinson patients (n = 117)	Controls (n = 156)		
**Age (year)**	61.66 ± 11.28	61.86 ± 6.29	-	-
**Weight (kg)**	75.58 ± 16.17	71.35 ± 11.39	-	-
**Height (cm)**	171.23 ± 8.35	174.87 ± 9.15	-	-
**BMI (kg/m** ^**2**^ **)**	25.26 ± 5.41	24.88 ± 4.14	0.43	0.07
**Physical activity level**	6.29 ± 0.70	7.90 ± 1.07	< 0.001	1.78
**Right 2D:4D**	1.02 ± 0.03	1.01 ± 0.07	0.12	0.18
**Left 2D:4D**	1.02 ± 0.02	1.01 ± 0.05	0.40	0.26
**Mean 2D:4D**	1.02 ± 0.02	1.01 ± 0.05	0.18	0.26
**D**_**R−L**_ **2D:4D**	0.005 ± 0.02	-0.0006 ± 0.04	0.23	0.17
**HGS right hand (kg)**	30.71 ± 9.85	32.85 ± 6.07	0.02	0.26
**HGS left hand (kg)**	29.85 ± 9.46	31.73 ± 4.86	0.03	0.24
**Level of disability**	2.31 ± 0.69	-	-	-

Notes; BMI: body mass index; HGS: hand grip strength; D_R−L_ 2D:4D: right hand 2D:4D - left hand 2D:4D

**Fig. 2:**
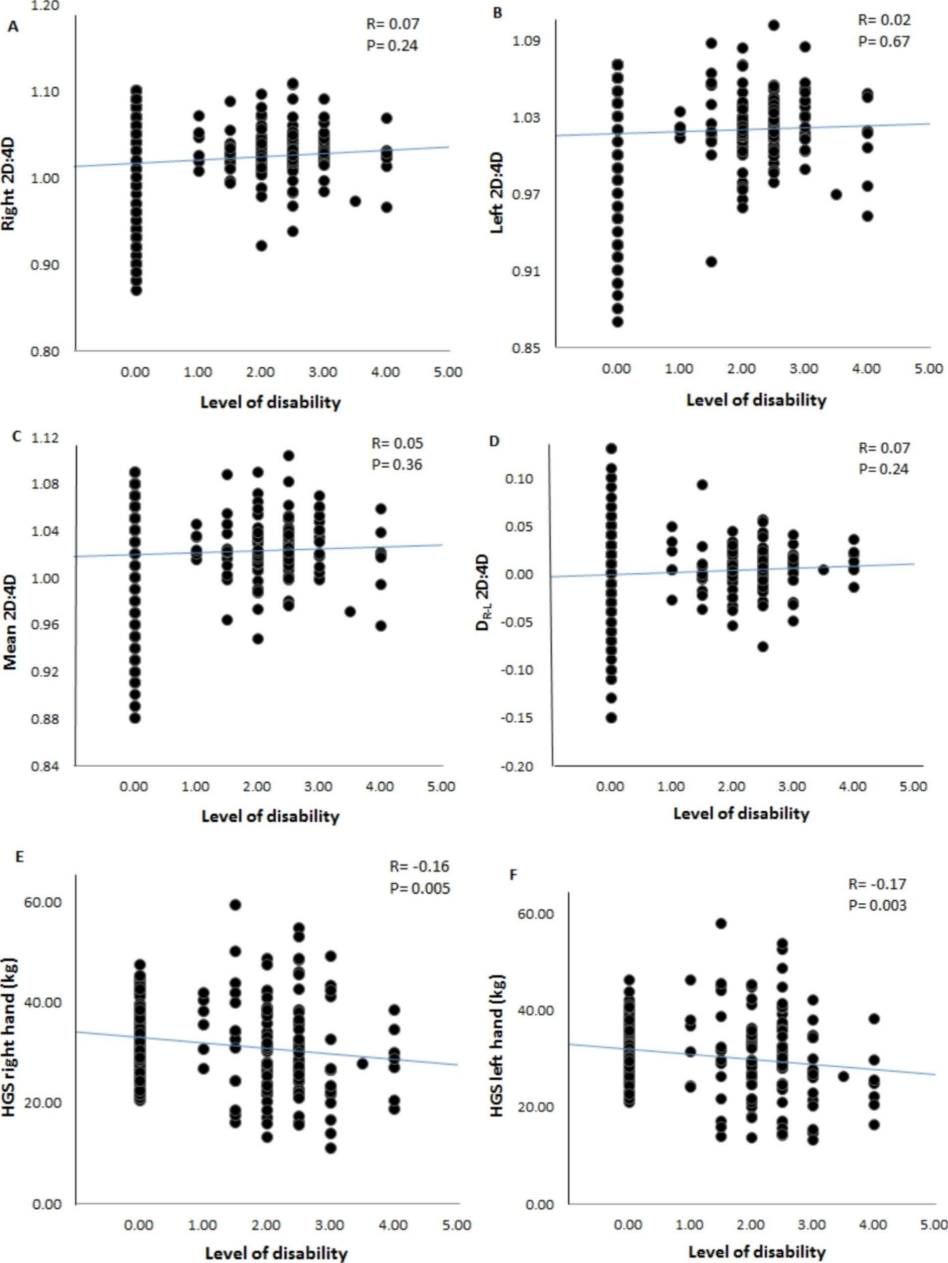
Associations between 2D:4D and HGS with the level of disability of Parkinson patients. Right 2D:4D (A), left 2D:4D (B), mean 2D:4D (C), D_R−L_ 2D:4D (D), HGS right hand (E) and HGS left hand (F) Notes; D_R−L_ 2D:4D: right hand 2D:4D - left hand 2D:4D; HGS: handgrip strength

The results of the correlation test showed a significant negative relationship between the Parkinson’s disease and right and left HGS (R = − 0.16, P = 0.005, R = − 0.17, P = 0.003; respectively, Fig. 2, E and F). In contrast, regarding the digit ratio, the results showed that no significant relationship was observed between Parkinson disease and 2D:4D of the right and left hand (R = 0.07, P = 0.24; R = 0.02, P = 0.67, respectively, Fig. 2, A and B). In addition to the ratio of the right and left 2D:4D, as shown in Fig. 2, there was no significant relationship between the mean ratio of the fingers and D_R−L_ 2D:4D with the Parkinson disease (R = 0.05, P = 0.36; R = 0.07, P = 0.24, respectively, Fig. 2, C and D).

As shown in Table 2, the regression results showed that the right and left HGS were not able to predict Parkinson’s disease (P = 0.25, P = 0.16; respectively).

**Table 2:** Multiple linear regression analysis with the level of disability of Parkinson patients as the dependent variable

Variable	B	β	SE	P	Durbin– Watson
Dependent variable: Level of disability (R^2^ = 0.036)					
**HGS left hand**	-0.019	-0.11	0.014	0.16	0.30
**HGS right hand**	-0.014	-0.09	0.013	0.25	

Notes; HGS: hand grip strength

In addition, the result of Table 3 showed significant negative correlation between the right and left HGS with D_R−L_ 2D:4D in the Parkinson group (R = -0.18, P = 0.04; R = -0.19, P = 0.03; respectively). Right HGS showed significantly negative correlation with right and left 2D:4D, D_R−L_ 2D:4D and mean 2D:4D in the control group (R = -0.80, P < 0.001; R = -0.48, P < 0.001; R = -0.60, P < 0.001; R = -0.71, P < 0.001; respectively). Also, left HGS showed a significant negative correlation with right and left 2d:4d and mean 2D:4D in control group (R = -0.47, P < 0.001; R = -0.68, P < 0.001; R = -0.61, P < 0.001; respectively).

**Table 3:** Relationship between HGS with 2D:4D

	Right HGS				left HGS			
	Parkinson		Control		Parkinson		Control	
	R	P	R	P	R	P	R	P
**Right 2D:4D**	-0.10	0.27	-0.80	**< 0.001**	-0.16	0.07	-0.47	**< 0.001**
**Left 2D:4D**	-0.04	0.61	-0.48	**< 0.001**	-0.01	0.88	-0.68	**< 0.001**
**D**_**R−L**_ **2D:4D**	-0.18	**0.04**	-0.60	**< 0.001**	-0.19	**0.03**	0.08	0.32
**Mean 2D:4D**	-0.03	0.7	-0.71	**< 0.001**	-0.10	0.27	-0.61	**< 0.001**

Notes; HGS: hand grip strength; D_R−L_ 2D:4D: right hand 2D:4D - left hand 2D:4D

## Discussion

This study aimed at investigating the relationship between Parkinson disease and HGS and 2D:4D ratio in older males. The results showed a significant negative relationship between Parkinson disease and right and left HGS, but in contrast, there was no significant relationship between Parkinson disease with 2D:4D right and left hand, mean finger ratio and D_R−L_ 2D:4D.

The cause of Parkinson’s disease is unknown; genetic and environmental factors can be among the causes of this disease [2, 3, 39]. The hypothesis that early life factors may contribute to Parkinson’s disease in later life is intriguing. Prenatal and postnatal periods are a critical time for brain development, during which the generation, migration, and proliferation of neurons are completed and the basic structure of the brain is established [40]. Sex hormones are the most important drivers of structural and functional sexual differentiations in the brain, while they are also a major cause of sex differences in disease susceptibility [41]. Studies have shown that hormone levels in adulthood play a role in increasing the incidence of Parkinson disease in men [42] and there was also evidence for the role of estrogen as a neuroprotector and its effects on dopaminergic function [43].

The results showed that the right and left HGS is significantly associated with Parkinson disease. In this regard, a study by Roberts et al. on the relationship between HGS and the severity and duration of Parkinson disease showed that an increase in the severity is associated with a decrease in HGS. Their results showed the effect of Parkinson on muscle strength; they stated that although the mechanism is unclear, it seems to be independent of the duration of the disease [44]. Increased severity of Parkinson in both the Unified Parkinson Disease rating scale and the Hoehn and Yahr scale is associated with weaker compression strength (regardless of the duration of the disease) [44]. In addition, the results of a study by Jones et al. showed that HGS is associated with upper and lower body muscle characteristics in people with Parkinsonwhile, in healthy people of the same age, mobility indicates muscle activity [15]. The main cause of muscle weakness in Parkinson is not known, but most likely, it indicates dopamine degradation in the striatum and movements of the cerebral cortex, leading to abnormal muscle activation. Poor compression strength may reflect poor muscle activation during daily life, resulting in impaired motor cortex ability to activate spinal motor neurons, slower onset of voluntary muscle contractions, or impaired motor function [45, 46]. Reduction nigro-striatal dopamine in Parkinson causes increased tonic inhibition of the thalamus and thus reduced motor cortex stimulation indicating that this can disrupt the corticospinal activation of muscle and abnormal electromyographic (EMG) activation patterns have been described in Parkinson’s disease throughout isometric movements in keeping with impaired muscle activation [11, 47].

The results of the study on the relationships between Parkinson and the digit ratio of the right hand, left hand, mean finger ratio and D_R−L_ 2D:4D indicated that there was no significant relationship. Studies have shown that a decrease in the 2D:4D ratio is associated with an increase in the fetal testosterone levels relative to the fetal estrogen levels. In contrast, high values ​​of the digit ratio are associated with higher levels of fetal estrogen than fetal testosterone [30]. As a result, sexual traits can have an acceptable relationship with digit ratios [30, 34]. Sex difference is strongly expressed on the right hand [48]. The ratio of fingers is between two distinct ranges: individuals with a digit ratio greater than one are defined as a dove type, and individuals with a digit ratio of less than one are defined as a hawk type. It can be said that the proportion of men fingers is lower than women and indicates a high level of testosterone [34]. The best relationship with 2D:4D was shown among the men. Previous studies have shown a direct relationship between male 2D:4D and testosterone-dependent traits during puberty and adulthood [49]. In addition, the D_R−L_ 2D:4D and D_L−R_ 2D:4D are indicators that may indicate 2D:4D asymmetry between the left and right hands. Previous evidence suggests that the 2D:4D in the right hand and the D_R−L_ 2D:4D were inversely related to prenatal exposure and sensitivity to testosterone. Reports indicated that the D_R−L_ 2D:4D is more clearly associated with exposure or sensitivity to prenatal estrogen than 2D:4D in the right hand [50].

As mentioned, sex is the most important factor in the development and progression of Parkinson disease. Estrogens have neuroprotective effects on the nigrostriatal dopaminergic system and can modulate monoamine oxidase (MAO). Moreover, the neuroprotective effects of estrogens may be inhibited by interfering with the production of interleukin-6 and increasing inflammation, and play an important role in the development and progression of Parkinson disease [51, 52]. In addition, some studies have shown that modulation of the neuroinflammatory response by estrogen is involved in neuroprotective effects, and other studies have shown that neuroinflammation and microglial activation play an important role in Parkinson progression [51, 53].

Studies have shown that digit ratio can indirectly show the rate of fetal development during the fetal period in the uterus and the changes that occur during this period (any changes in the uterine environment such as malnutrition or hormonal changes not only changes the structure of systems and organs of the fetus, but also affects the ratio of the fingers) [25]. Thus, the prenatal period is defined as the period during which the endocrine glands, environment, and behaviors may all play a role in increasing the risk of disease [54]. Regarding neurological diseases, a high 2D:4D ratio is associated with an increased risk of Alzheimer’s disease in men, while a decreased 2D:4D ratio is associated with an increased risk of the disease in women [55]. Furthermore, a decrease in 2D:4D is associated with an increased risk of amyotrophic lateral sclerosis [56]. In addition, Kobus et al. stated that depending on sex, a different proportion of prenatal sex steroids may be a risk factor for migraine in adults. Men with migraine were likely be exposed to higher levels of estrogen than testosterone during prenatal life [57]. The results of Collinson et al. on the relationship between 2D:4D and schizophrenia showed that there was no significant difference in mean finger length. However, the 2D:4D ratio was significantly different between patients and the control group. They stated that this effect was predominantly in men, consistent with the “less masculinised” pattern and the hypothesis that schizophrenia may be associated with disturbances in prenatal circulating testosterone [58].

The results of the research showed a significant negative relationship between the right and left HGS and 2D:4D of the participants of the control group. Also, in the Parkinson group, only HGS had a weak significant negative relationship with D_R−L_ 2D:4D. In addition, HGS was significantly different between the two control groups and the Parkinson group. Fink et al. stated that HGS was higher in men with less 2D:4D. They stated that HGS was strongly related to strength in other muscle groups, so they concluded that prenatal testosterone may have a primary organizing effect on strength in men, and this is probably more common in human groups [59]. Similarly, Kociuba et al. found that 2D:4D had a significant negative correlation with HGS [60]. Also, Pasanen et al. found a weak negative relationship between 2D:4D and HGS in their meta-analysis [61]. Muscle strength is a powerful marker of current health and a predictor of future health [61–66]. In adults, low HGS is significantly associated with early cardiovascular mortality and disability [63, 66]. Findings showed that people with low 2D:4D are healthier [20, 66–70]. This is the first study on Parkinson, and we have only studied men. Due to the sexual dimorphism in the digit ratio, the relationship between 2D:4D ratio, androgens and fetal estrogen is generally quite evident in men [31]. In this study, no relationship was observed between the digit ratio and the Parkinson disease. Therefore, it is possible that exposure to androgen and estrogen levels at different times can affect the risk of developing Parkinson disease and its progression. In addition, the 2D:4D ratio varies greatly in different geographical areas, sex, and races [71]. Moreover, 2D:4D may be different in people of the same ethnicity, probably because of different gene stores [72]. The results of the study do not support the hypothesis that 2D:4D ratio predicts Parkinson’s disease. In addition, due to the superiority of testosterone levels over estrogen in men, a decrease in the 2D:4D is often seen in them, and given the mechanism of Parkinson disease and the importance of estrogen levels,. it is suggested that future studies examine the relationship between digit ratio and Parkinson disease in different ethnicities, women and among a larger number of people.

## Conclusion

In general, the results showed a significant negative relationship between Parkinson disease and right and left HGS in elderly males. But, there was no evidence of a significant association between 2D:4D ratio and the Parkinson’s disease. Moreover, the results showed that the right and left HGS were not able to predict Parkinson disease in elderly males. Based on the results, increasing muscle strength is related to reducing the level of disability in Parkinson’s patients and prescribing appropriate physical activity to increase muscle strength can be beneficial for these patients. For more accurate conclusions, it is necessary to conduct research on more population, different sex and races and compare them with each other.

## Data Availability

The datasets used and analyzed during the current study are available from the corresponding author on reasonable request.

## Abbreviations

2D:4D: The second to fourth digit ratio
HGS: Handgrip strength
EMG: Electromyography
MAO: Monoamine oxidase
BMI: Body mass index
SD: Standard deviations
ANOVA: Analysis of variance
